# Effect of Disinfection Method and Testing Methodology on the Performance of a Breath-Enhanced Jet Nebulizer

**DOI:** 10.3390/pharmaceutics16010016

**Published:** 2023-12-21

**Authors:** Kanjanamala Agoramurthi, Ariel Berlinski

**Affiliations:** 1 Pulmonary and Sleep Medicine Division, Department of Pediatrics, University of Arkansas for Medical Sciences, Little Rock, AR 72205, USA; 2 Pediatric Aerosol Research Laboratory, Arkansas Children’s Research Institute, Little Rock, AR 72202, USA

**Keywords:** nebulizer performance evaluation, disinfection, cystic fibrosis, breath-enhanced nebulizer, drug output, solution output

## Abstract

National guidelines for cystic fibrosis recommend cleaning and disinfecting nebulizers after each use. We tested two groups of five reusable breath-enhanced nebulizers after 0, 5, 10, 15, 20, 30, 60, 90, 120, 150, and 180 sterilization (baby bottle sterilizer) or cleaning cycles. The nebulizers were operated for 7 min (6 L/min) after loading albuterol (2.5 mg/3 mL), and they were evaluated with and without breathing simulation after cleaning/sterilization (0–180 and 0–60 cycles, respectively). Over the course of 180 cleaning/sterilization cycles, the mean (SD) solution output was 1.33 mL (0.12 mL)/1.29 mL (0.08 mL); the nebulizer mass remaining in the nebulizer was 61.5% (5.2%)/63% (4%); sputtering time was 4.7 min (0.8 min)/4.8 s (0.6 min); inspiratory filter was 19% (3%)/18.5% (2.4%); expiratory filter was 6.7% (1.1%)/6.7% (0.8%); and difference in drug output calculated using the solution output and nebulizer mass was 6.8% (4%)/5.2% (2.9%). Thermal disinfection with a baby-bottle sterilizer did not alter the performance of a reusable breath-enhanced nebulizer. The nebulizer test performed without breathing simulation underestimated its performance. The calculation of the drug output based on the solution output resulted in its overestimation.

## 1. Introduction

Cystic fibrosis is an autosomal recessive disease caused by mutations in the cystic fibrosis transmembrane conductance regulator protein, a channel located at the surface of airway epithelial cells and sub-mucosal glands [1]. Though multiple systems in the body, including respiratory, digestive, and reproductive systems, can be affected, progressive pulmonary disease is the primary cause of morbidity and mortality [2].

National guidelines recommend the use of different inhaled medications to maintain and improve lung health [3]. These recommendations include hypertonic saline, dornase alfa, tobramycin, aztreonam, and albuterol. Previous reports have indicated that nebulizers used by people with cystic fibrosis (PwCF) could become infected if not properly disinfected [4,5,6,7,8,9,10,11,12]. Some authors have reported nebulizer contamination rates of up to 65% [4,5]. The use of contaminated nebulizers carries the risk of re-inhaling bacteria deeper into the lungs. The Cystic Fibrosis Foundation developed and published infection prevention guidelines that include recommendations for the cleaning and sterilization of nebulizers after each use [13,14]. The guidelines provide specific recommendations regarding cleaning, which include cleaning the nebulizer with soap and water, followed by rinsing it with water to remove the residual soap. Disinfection can be achieved by using either chemical- or thermal-based methods. Chemical-based methods include submersion in 70% isopropyl alcohol for 5 min or 3% hydrogen peroxide for 30 min. Chemical-based methods should be followed by rinsing the nebulizer with sterile water and air-drying its parts before storage. Thermal-based methods include the following: (1) submersion in boiling water and boiling for 5 min, (2) submersion in water in a microwave-safe receptacle and microwaving for 5 min, and (3) placing in the top rack of the dishwasher if the water temperature is equal to or higher than 70 °C or 158 °F for 30 min. Lately, the use of electric steam sterilizers in respiratory devices has become popular and has also been recommended by the Cystic Fibrosis Foundation as a thermal-based method [14,15,16,17]. Several studies have shown the effectiveness of an electric steam sterilizer in killing microorganisms relevant to cystic fibrosis such as *methicillin resistant Staphylococcus aureus*, *Burkholderia cepacia*, *Haemophilus influenzae*, *mucoid* and *non-mucoid Pseudomonas aeruginosa*, *Stenotrophomonas maltophilia*, and *non-tuberculous mycobacteria* [15,16,17]. The low disinfection rates of nebulizers have been correlated with the greater recovery of bacteria in nebulizers used by PwCF [18]. Recent surveys have shown that boiling is the most common method used by PwCF and their families [19,20]. The frequency of disinfection has been reported as significantly lower than recommended in many studies [19,20,21]. This behavior was reported to be exacerbated while traveling [22].

There are different types of nebulizers available for the delivery of medications used by PwCF. These include jet nebulizers, ultrasonic nebulizers, and mesh nebulizers [23,24,25]. The latter are more efficient, faster, and significantly more expensive. Many of them have been optimized for the delivery of specific drugs and have been approved by regulatory agencies as a drug/device combination (i.e., Cayston and Altera devices). Ultrasonic nebulizers were used more than three decades ago to deliver inhaled tobramycin [26]. However, they are no longer favored because they can denature proteins and are not effective in delivering budesonide, among other reasons [23,27]. Jet nebulizers are the most used devices by PwCF. There are different types of jet nebulizers [23,24]. The continuous-output jet nebulizer produces and releases aerosols during inspiration and expiration. This feature results in environmental exposure and significant drug waste. These devices are inexpensive and are typically used to deliver albuterol. Breath-enhanced nebulizers incorporate an inspiratory one-way valve, resulting in increasing drug delivery and decreasing environmental exposure [23,24,25]. The PARI LC Jet nebulizer (PARI Respiratory Inc., Midlothian, VA, USA), a device from the latter category, was approved by the Food and Drug Administration as a drug/device combination for the delivery of dornase alfa, tobramycin, and budesonide [28,29,30]. This nebulizer was also used in hypertonic saline trials [31]. A recent survey showed that PARI LC Plus was the most used nebulizer by PwCF in the United States [19]. Breath-actuated nebulizers only produce aerosols during inhalation, thus resulting in minimal environmental exposure. Several studies have highlighted that devices cannot be used interchangeably [32,33].

The evaluation of the effects of repeated cleaning and disinfection on nebulizer performance has yielded conflicting results [34,35]. Standaert et al. did not find differences in performance when evaluating disposable units [34]. The limitations of their study included not using breathing simulation and performing a limited number of disinfection cycles (10) with a dishwasher. Collins et al. reported a deterioration of nebulizer performance when thermal-based methods of disinfection were used [35]. While this study increased the number of disinfection cycles to 60, it did not include breathing simulation in the assessment of nebulizer performance. Another methodological limitation is the fact that they used solution output as a proxy of drug output, thus overestimating drug output [36,37]. Both studies likely underestimated drug output by not using simulated breathing [38].

The method used to evaluate performance is crucial to achieving valid conclusions. O’Callaghan et al. previously demonstrated that the use of weight variation before and after nebulization results in the overestimation of drug output [36]. Tandon et al., using radiolabeled aerosols, also found that the gravimetric method overestimated drug output [37]. Barry et al. demonstrated that the type of breathing simulation impacts drug output results [38]. Thus, proper evaluation of nebulizer performance is conducted using specific breathing patterns recommended by international organizations [39].

Given the limited available data, the concerns about methodological aspects of the available studies, the recommendation of sterilization after each use, and the fact that this breath-enhanced nebulizer is the most used by PwCF, a re-evaluation of the effect of thermal disinfection of these nebulizers was warranted.

In this study, we aimed to compare the nebulizer performance of reusable breath-enhanced jet nebulizers cleaned or cleaned and disinfected with a baby bottle sterilizer with and without breathing simulation. We hypothesized that a thermal-based method of disinfection will not affect nebulizer performance. We hypothesized that evaluating nebulizer performance without breathing simulation of a breath-enhanced device will result in its underestimation. Finally, we also hypothesized that using the solution as a surrogate for drug output will result in its overestimation.

## 2. Methods

The study was performed at the Pediatric Aerosol Research Laboratory at Arkansas Children’s Research Institute, Little Rock, AR, USA.

### 2.1. Nebulizers and Sterilization

Ten units of a reusable breath-enhanced nebulizer (PARI LC Plus, PARI Respiratory Equipment, Midlothian, VA, USA) were tested. Nebulizers were connected to a 0–15 L/min back-compensated flowmeter (Timeter, Allied Healthcare, St. Louis, MO, USA) and operated with air at 6 L/min for 7 min similarly to previous publications [35,40]. Nebulizers were only operated during the scheduled evaluation.

The sterilization process was as follows: the baby bottle sterilizer (Philips Avent iQ24 steam sterilizer, Andover, MA, USA) was filled with 90 mL of double-distilled water, the nebulizers were disassembled and placed inside the chamber, and run for 6 min per manufacturer’s recommendations. Upon completion of a sterilization cycle, the nebulizers were cooled down to room temperature before starting the next cycle. The cleaning process included rinsing the nebulizers with double-distilled water followed by air-drying.

### 2.2. Study Design

The units were equally divided into two groups: cleaning with water (control) and sterilization with baby bottle sterilizer. Nebulizer performance with and without breathing simulation was evaluated before the first cycle of cleaning/sterilization and after 5, 10, 15, 20, 30, and 60 cycles (Table 1). Nebulizer performance with breathing simulation was continued after 90, 120, 150, and 180 cycles (Table 1). The number of cycles represents daily cleaning/sterilization for the life of the reusable nebulizer (6 months).

### 2.3. Testing Procedure

**Figure 1:**
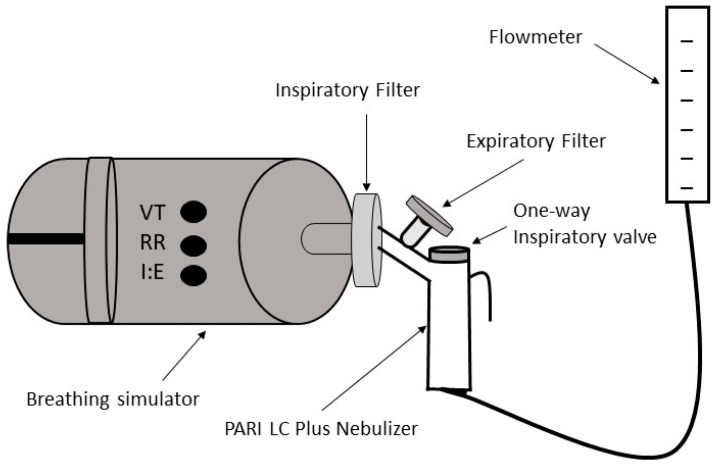
Experimental set up. VT = tidal volume. RR = respiratory rate. I:E = inspiratory:expiratory ratio.

Laboratory temperature (°C) and humidity (%) were recorded for every test. Nebulizers were weighed dry and after loading albuterol solution (2.5 mg/3 mL, (RiteDose Pharmaceuticals, Columbia, SC, USA). Nebulizers were connected in series to an adapter with expiratory and inspiratory filters, and to a breathing simulator (Harvard Apparatus 613 Dual Phase Respirator Pump, Harvard Apparatus, Holliston, MA, USA) (Figure 1) [33,38]. The breathing simulator allows independent adjustment of tidal volume, respiratory rate, and inspiratory:expiratory ratio. The breathing simulator was set up with a tidal volume of 500 mL, inspiratory:expiratory ratio of 1:1, and a respiratory rate of 15 breaths/min according to international standards for evaluation of nebulizers [39]. The accuracy of nebulizer flow and breathing simulator parameters were verified before each experiment with a flow analyzer (Certifier Plus Flow Analyzer, TSI Inc., Shoreview, MN, USA). Single-use filters (PARI Respiratory Equipment, Midlothian, VA, USA) were changed with every experiment. Upon completion of each experiment, inspiratory and expiratory filters were placed in 50 mL tubes. In total, 10 mLs of double-distilled water was added and the tubes were vortexed. The nebulizers were weighed after completing 7 min of operation, and after adding 10 mL of double-distilled water. Biochemical determination of albuterol concentration was measured via spectrophotometry.

### 2.4. Biochemical Determination

Serial dilutions of a sample of known albuterol concentration were used to create a calibration curve in a spectrophotometer (ThermoScientific, Biomate 160, Thermo Fisher Scientific, Waltham, AM, USA). The absorbance peak was measured at 276 nm. The calibration curve was linear up to 280 µg/mL with an R^2^ of 0.99. The proper functioning of the device was verified daily with samples of different known concentrations. Samples obtained from the nebulizer and filter washings were placed in 1 mL disposable cuvettes and the absorbance was measured at 276 nm. 

### 2.5. Outcome Variables

The following variables were evaluated: solution output (mL, loaded nebulizer weight—post-nebulization weight), sputtering time (beginning of erratic aerosol production), nebulizer mass (mass remaining in the nebulizer upon completion of nebulization expressed as percentage of loading mass = albuterol concentration × solution volume after adding 10 mL of double-distilled water upon completion of nebulization), and inspiratory and expiratory filter expressed as percentage of loading dose. We also compared drug output assessed by solution output (solution output × 833 µg/mL) and drug output assessed by mass released (2500 µg—nebulizer mass) and its difference. The mass balance of the recovered albuterol expressed as a percentage of the loading dose was calculated as quality control.

### 2.6. Statistical Analysis

Pairwise comparison between cleaning and sterilization after each of the established times was conducted with a Mann–Whitney U test. Pairwise comparison between breathing simulation and no breathing simulation after each of the established times was conducted with a Wilcoxon signed rank test. Comparison of variables across the study (0–180 cycles) was conducted with a Friedman test followed by Dunn’s test for multiple comparisons when required. A *p* value < 0.05 was considered statistically significant. A statistical software package was used for all the calculations (Prism 9.51 GraphPad Software, La Jolla, CA, USA).

## 3. Results

Results from each outcome variable (solution output, sputtering time, nebulizer mass, inspiratory filter, expiratory filter, and drug output calculated by solution output and mass released) were reported without and with breathing simulation followed by comparisons between cleaning and disinfection and between testing with and without breathing simulation. 

### 3.1. Quality Assurance

Median (99% CI) temperature and humidity for all experiments were 19.6 °C (19.3–19.8 °C) and 69.3% (65.1–71.4%), respectively. Median (95% CI) mass balance was 87.1% (86.5–87.6%), 86.7% (85.6–87.6%), and 87.5% (86.6–88.4%) for both groups together, cleaning group alone, and sterilization group alone, respectively.

### 3.2. Solution Output

#### 3.2.1. Evaluation without Breathing Simulation

**Figure 2:**
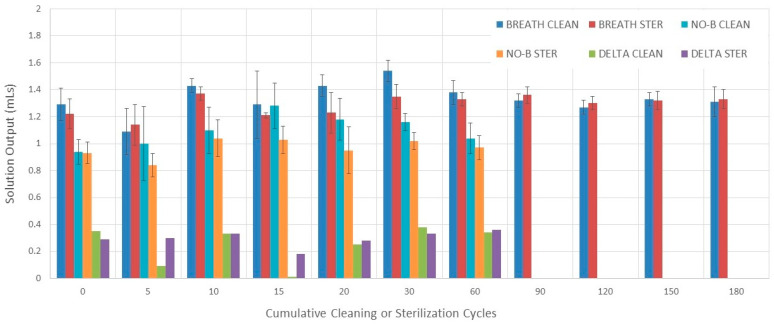
Solution output. Bars represent the mean value and error bars represent 1 SD. BREATH CLEAN = values obtained with breathing simulation after cleaning. BREATH STER = values obtained with breathing simulation after sterilization. NO-B CLEAN = values obtained without breathing simulation after cleaning. NO-B STER = values obtained without breathing simulation after sterilization. DELTA CLEAN = difference between values obtained with and without breathing simulation after cleaning. DELTA STER = difference between values obtained with and without breathing simulation after sterilization.

No differences in solution output among 0–60 cleaning cycle measurements were found during evaluation without breathing simulation (*p* = 0.006, but not significant after multiple comparisons analysis) (Figure 2). The solution output varied among 0–60 sterilization cycle measurements during evaluation without breathing simulation (*p* = 0.009). The solution output after 30 sterilization cycles was higher than after 0 and 5 sterilization cycles (*p* = 0.016 and *p* = 0.016, respectively).

#### 3.2.2. Evaluation with Breathing Simulation

The solution output varied among 0–180 cleaning cycle measurements during evaluation with breathing simulation (*p* = 0.002) (Figure 2 and Table 2). The solution output after 5 cleaning cycles was lower than after 30 cycles (*p* = 0.005). The solution output after 30 cleaning cycles was higher than after 120 cleaning cycles (*p* = 0.016). The solution output varied among 0–180 sterilization cycle measurements during evaluation with breathing simulation (*p* = 0.0009). However, none of these differences were significant after multiple comparisons analysis.

#### 3.2.3. Comparisons

There was no difference in solution output between the cleaning and sterilization groups during evaluation with breathing simulation at each cumulative cycle point (0–180) except after 20 and 30 cycles where the cleaning group was higher (*p* = 0.032 and *p* = 0.005, respectively) (Figure 2).

The solution output measured with breathing simulation was higher than the one measured without breathing simulation at each measuring point between 0 and 60 cycles of cleaning or sterilization. The only exception was after five cleaning cycles (*p* = 0.58) (Figure 2).

The mean (SD) solution output over 180 cleaning and sterilization cycles was 1.33 mL (0.12 mL) and 1.29 mL (0.08 mL), respectively. The mean (SD) differences in solution output between measurements conducted with and without breathing simulation for the cleaning and sterilization groups were 0.32 mL (0.14 mL) and 0.32 mL (0.06 mL), respectively.

### 3.3. Nebulizers Mass

#### 3.3.1. Evaluation without Breathing Simulation

**Figure 3:**
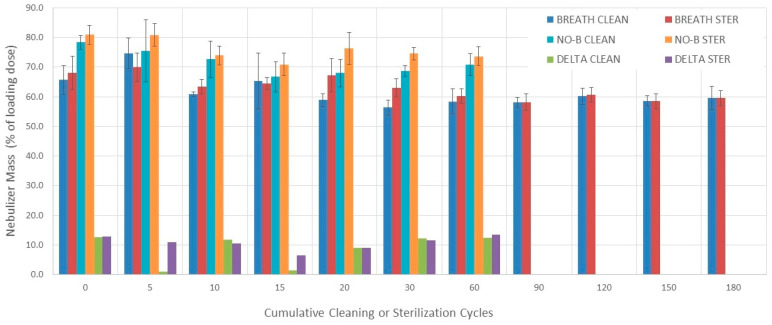
Nebulizer mass. Bars represent the mean value and error bars represent 1 SD. BREATH CLEAN = values obtained with breathing simulation after cleaning. BREATH STER = values obtained with breathing simulation after sterilization. NO-B CLEAN = values obtained without breathing simulation after cleaning. NO-B STER = values obtained without breathing simulation after sterilization. DELTA CLEAN = Difference between values obtained without and with breathing simulation after cleaning. DELTA STER = Difference between values obtained without and with breathing simulation after sterilization.

Nebulizer mass varied among 0–60 cleaning cycle measurements during evaluation without breathing simulation (*p* = 0.0009) (Figure 3). A decrease in nebulizer mass from 0 to 15 cleaning cycles was noticed (*p* = 0.016). The nebulizer mass varied among 0–60 sterilization cycle measurements during evaluation without breathing simulation (*p* = 0.005). A decrease in nebulizer mass from 0 and 5 to 15 sterilization cycles was found (*p* = 0.027 and *p* = 0.027, respectively).

#### 3.3.2. Evaluation with Breathing Simulation

The nebulizer mass varied among 0–180 cleaning cycle measurements during evaluation with breathing simulation (*p* = 0.0012) (Figure 3 and Table 2). The nebulizer mass was higher after 0 cleaning cycles than after 30 cleaning cycles (*p* = 0.033). The nebulizer mass was higher after 5 cleaning cycles than after 30 and 90 cleaning cycles (*p* = 0.033 and *p* = 0.002, respectively). The nebulizer mass varied among 0–180 sterilization cycle measurements during evaluation with breathing simulation (*p* < 0.0001). The nebulizer mass decreased from 0 to 90 and 150, 5 to 90 and 150, and 20 to 90 sterilization cycles (*p* = 0.023, *p* = 0.047, *p* = 0.002, *p* = 0.002, *p* = 0.005, and *p* = 0.047, respectively).

#### 3.3.3. Comparisons

There was no difference in nebulizer mass between cleaning and sterilization groups during evaluation with breathing simulation at each cumulative cycle point (0–180) except after 20 and 30 cycles where the sterilization group was higher (*p* = 0.016 and *p* = 0.006, respectively) (Figure 3). 

The nebulizer mass measured with breathing simulation was lower than the one measured without breathing simulation at each measuring point between 0 and 60 cycles of cleaning or sterilization. The only exceptions were after 5 and 15 cleaning cycles (*p* = 0.86 and *p* = 0.77, respectively) (Figure 3).

The mean (SD) nebulizer mass over 180 cleaning and sterilization cycles during evaluation with breathing simulation was 61.5% (5.2%) and 63% (4%), respectively. The mean (SD) differences in nebulizer mass between measurements conducted with and without breathing simulation for the cleaning and sterilization groups were 11.3% (5.2%) and 11.4% (2.4%), respectively.

### 3.4. Sputtering Time

#### 3.4.1. Evaluation without Breathing Simulation

**Figure 4:**
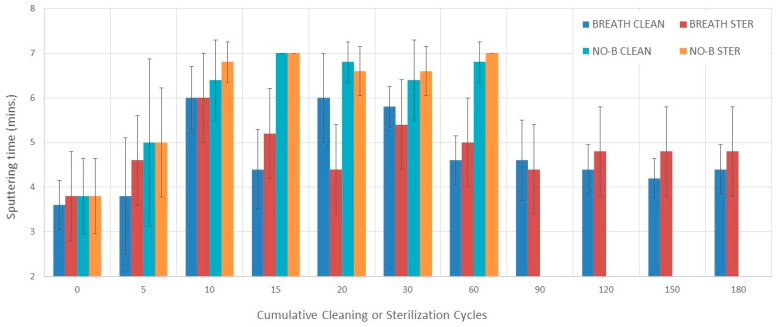
Sputtering time. Bars represent the mean value and error bars represent 1 SD. BREATH CLEAN = values obtained with breathing simulation after cleaning. BREATH STER = values obtained with breathing simulation after sterilization. NO-B CLEAN = values obtained without breathing simulation after cleaning. NO-B STER = values obtained without breathing simulation after sterilization.

There were no statistically significant differences in sputtering time among 0–60 cleaning and sterilization cycles during evaluation without breathing simulation (Figure 4).

#### 3.4.2. Evaluation with Breathing Simulation

Sputtering times varied among 0–180 cleaning and sterilization cycle measurements during evaluation with breathing simulation (*p* = 0.0003 and *p* = 0.013, respectively) (Figure 4 and Table 2). Sputtering time was longer after 10 cleaning and sterilization cycles than at 0 cycles (*p* = 0.047 and *p* = 0.033, respectively).

#### 3.4.3. Comparisons

There was no difference in sputtering between the cleaning and sterilization groups during evaluation with breathing simulation at each cumulative cycle point (0–180) except after 20 and 30 cycles when the sterilization group was shorter than the cleaning group (*p* = 0.02 and *p* = 0.03, respectively) (Figure 4).

The mean (SD) sputtering time over 180 cleaning and sterilization cycles during evaluation with breathing simulation was 4.7 min (0.8 min) and 4.8 min (0.6 min), respectively. The mean (SD) differences in sputtering time between measurements conducted with and breathing simulation for the cleaning and sterilization groups were 1.2 min (0.9 min) and 1.8 min (0.8 min), respectively.

### 3.5. Inspiratory Filter

#### 3.5.1. Evaluation without Breathing Simulation

**Figure 5:**
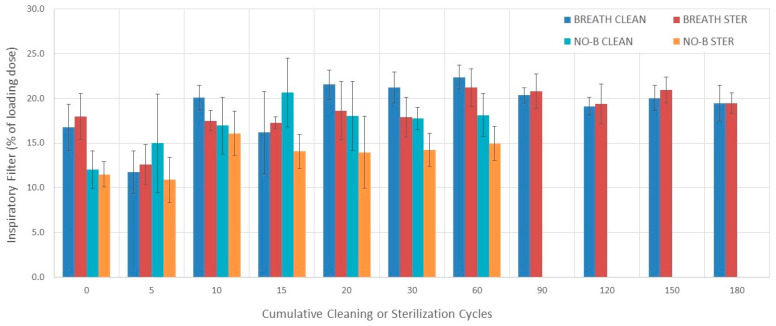
Inspiratory filter. Bars represent the mean value and error bars represent 1 SD. BREATH CLEAN = values obtained with breathing simulation after cleaning. BREATH STER = values obtained with breathing simulation after sterilization. NO-B CLEAN = values obtained without breathing simulation after cleaning. NO-B STER = values obtained without breathing simulation after sterilization.

The inspiratory filter varied among 0–60 cleaning cycle measurements during evaluation without breathing simulation (*p* = 0.044) (Figure 5). An increase in inspiratory filter was found from 0 to 15 cleaning cycles (*p* = 0.015). The inspiratory filter varied among 0–60 sterilization cycle measurements during evaluation without breathing simulation (*p* = 0.006). An increase in inspiratory filter was found between 0 and 10 sterilization cycles (*p* = 0.016).

#### 3.5.2. Evaluation with Breathing Simulation

The inspiratory filter varied among 0–180 cleaning cycle measurements during evaluation with breathing simulation (*p* = 0.003) (Figure 5 and Table 2). The inspiratory filter increased from 5 to 20 and 60, and from 15 to 60 cleaning cycles (*p* = 0.023, *p* = 0.002, and *p* = 0.023, respectively). The inspiratory filter varied among 0–180 sterilization cycle measurements during evaluation with breathing simulation (*p* < 0.0001). The inspiratory filter increased from 5 to 20, 60, 90, and 150 sterilization cycles (*p* = 0.002, *p* = 0.009, *p* = 0.009, and *p* = 0.016, respectively).

#### 3.5.3. Comparisons

There was no difference in the inspiratory filter between the cleaning and sterilization groups during evaluation with breathing simulation at each cumulative cycle point (0–180) except after 10 and 30 cycles where the sterilization group was lower than the cleaning group (*p* = 0.02 and *p* = 0.031, respectively) (Figure 5). 

The inspiratory filter measured with breathing simulation was higher than the one measured without breathing simulation at each measuring point between 0 and 60 sterilization cycles. However, the difference was not statistically significant after 10 sterilization cycles (*p* = 0.37). The inspiratory filter measured with breathing simulation was higher than the one measured without breathing simulation at each measuring point between 0 and 60 cleaning cycles except after 5 and 15 cleaning cycles (*p* = 0.59 and *p* = 0.17, respectively) (Figure 5).

The mean (SD) inspiratory filter over 180 cleaning and sterilization cycles was 19% (3%) and 18.5% (2.4%), respectively. The mean (SD) differences in the inspiratory filter between measurements conducted with and breathing simulation for the cleaning and sterilization groups were 3.7% (3.8%) and 4.9% (2%) percentage points, respectively.

### 3.6. Expiratory Filter

#### 3.6.1. Evaluation without Breathing Simulation

**Figure 6:**
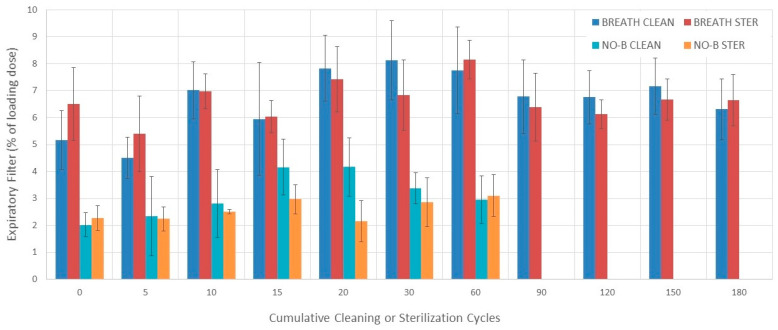
Expiratory filter. Bars represent the mean value and error bars represent 1 SD. BREATH CLEAN = values obtained with breathing simulation after cleaning. BREATH STER = values obtained with breathing simulation after sterilization. NO-B CLEAN = values obtained without breathing simulation after cleaning. NO-B STER = values obtained without breathing simulation after sterilization.

No differences in expiratory filter among 0–60 cleaning or sterilization cycle measurements were found during evaluation without breathing simulation (*p* = 0.60 and *p* = 0.08, respectively) (Figure 6).

#### 3.6.2. Evaluation with Breathing Simulation

The expiratory filter varied among 0–180 cleaning cycle measurements during evaluation with breathing simulation (*p* = 0.02) (Figure 6 and Table 2). The expiratory filter increased from 5 to 20 and 30 and from 0 to 30 cleaning cycles (*p* = 0.047, *p* = 0.01, and *p* = 0.033, respectively). The expiratory filter varied among 0–180 sterilization cycle measurements during evaluation with breathing simulation (*p* = 0.031). The expiratory filter increased from 5 to 60 sterilization cycles (*p* = 0.033).

#### 3.6.3. Comparisons

There was no difference in the expiratory filter between the cleaning and sterilization groups during evaluation with breathing simulation at each cumulative cycle point (0–180) (Figure 6).

The expiratory filter measured with breathing simulation was higher than the one measured without breathing simulation at each measuring point between 0 and 60 sterilization and cleaning cycles Figure 6).

The mean (SD) expiratory filter over 180 cleaning and sterilization cycles was 6.7% (1.1%) and 6.7% (0.8%), respectively. The mean (SD) differences in the expiratory filter between measurements conducted with and breathing simulation for the cleaning and sterilization groups were 4.4% (1.2%) and 4.8% (0.9%) percentage points, respectively [19,34,35].

### 3.7. Drug Output

#### 3.7.1. Evaluation without Breathing Simulation

The mean (SD) drug output calculated by measuring solution output and mass released from the nebulizer after 60 cleaning cycles was 36.7% (3.9%) and 28.5% (4.2%), respectively. The mean (SD) drug output calculated by measuring solution output and mass released from the nebulizer after 60 sterilization cycles was 32.3% (2.4%) and 24.1% (3.8%), respectively. The mean (SD) differences in drug output calculated by solution output and nebulizer mass were 8.2% (1.6%) and 8.2% (2.2%) after cleaning and sterilization, respectively. The mean and median (95% CI) of overestimation of using the solution output method after cleaning and disinfection was 29.7% and 28.3% (18.6–44.6%) and 35.7% and 33.4% (18.1–62.6%), respectively.

#### 3.7.2. Evaluation with Breathing Simulation

**Figure 7:**
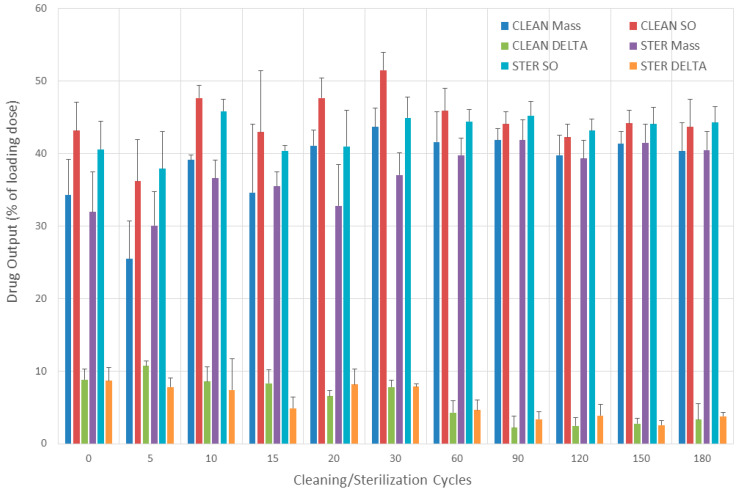
Drug output. Bars represent the mean value. CLEAN Mass = drug output calculated from nebulizer mass (after cleaning). STER Mass = drug output calculated from nebulizer mass (after sterilization). CLEAN SO = drug output calculated from solution output (after cleaning). STER SO = drug output calculated from solution output (after sterilization). CLEAN DELTA = CLEAN SO − CLEAN Mass. STER DELTA = STER SO − STER Mass.

Drug output results obtained with two different methods after cleaning and sterilization (0–180 cycles) and tested with breathing simulation can be seen in Figure 7. The mean (SD) drug output calculated by measuring solution output and mass released from the nebulizer after cleaning was 44.5% (3.8%) and 37.7% (5.4%), respectively. The mean (SD) drug output calculated by measuring solution output and mass released from the nebulizer after sterilization was 42.9% (2.5%) and 37.7% (3.9%), respectively. The mean (SD) difference in drug output calculated by solution output and nebulizer mass was 6.8% (4%) and 5.2% (2.9%) after cleaning and sterilization, respectively. The mean and median (95% CI) of overestimation of using the solution output method after cleaning and disinfection was 19.5% and 17.6% (7.1–25.3%) and 14.5% and 11.5% (8.3–24.9%), respectively.

#### 3.7.3. Comparisons

The overestimations of drug output when using solution output instead of measuring mass released and not using breathing simulation were 1.37 and 1.23-fold larger for the nebulizers under 60 cycles of cleaning or sterilization, respectively, than when breathing simulation was used.

## 4. Discussion

We compared the performance of reusable breath-enhanced nebulizers after undergoing either cleaning or thermal disinfection with a baby bottle sterilizer. We found that performance was essentially similar in both groups. We noticed an initial period of variable performance that stabilized after 30 cleaning or sterilization cycles. We also found that testing these nebulizers without breathing simulation resulted in underestimating their true drug output. Finally, we found that using solution output to calculate drug output resulted in its overestimation.

Our data agree with Standaert et al. who found no difference in the solution output of albuterol solution after 60 runs when operating a PARI LC jet nebulizer that was not sterilized without using a breathing simulation. They also showed an initial performance variability that stabilized after 30 runs. We found that the variability in output occurred until 60 cycles of cleaning or disinfection were completed. This represents a total of seven nebulizer runs conducted at 0, 5, 10, 15, 20, 30, and 60 cleaning disinfection cycles. Standaert et al. found an increase in solution output after 10 disinfection cycles with a dishwasher machine. This matches our data when comparing 0 to 10 disinfection cycles (Figure 2). Our evaluation was extended to 180 sterilization cycles and did not show deterioration of solution output over time. This contrasts with a report by Collins et al., who compared the solution output of PARI LC jet nebulizers without using breathing simulation. They evaluated the effect of different disinfections on the solution output of 0.9% saline solution. They reported a decline in solution output over time when thermal-based methods of disinfection were used. One difference between theirs and our study was that we did not use the nebulizer except during the scheduled evaluation times. Another difference is that they powered the nebulizers with a compressor, but we conducted it with central air, thus avoiding any potential deterioration of compressor performance [41]. This methodological decision was made with the intent of making the disinfection method the only variable of the study.

The nebulizer mass measured in our study (61.5–63%) was similar to the values reported by previous studies [24,42]. Rau et al. reported a nebulizer mass of 62.5% when testing a PARI LCD nebulizer [24]. Dennis reported 62–64% mass left in the PARI LC Plus nebulizers previously loaded with 3 mLs of 1% sodium fluoride solution [42].

Inspiratory filter data (19%) were slightly lower than those published by Dennis (25%) [42]. Similarly, expiratory filter data (6.7%) were lower than those published by Dennis (11.5%). However, the ratio between inspiratory and expiratory filter values was similar for both studies. Experimental design differences might account for differences between both studies since he used a larger tidal volume (635 mL), did not specify inspiratory time, and used compressors to power the nebulizer [42]. Inspiratory filter data were similar to a previous study (18.8%) from our own laboratory using the same nebulizer, similar tidal volume and respiratory rate but shorter inspiratory time [33]. In that study, nebulizers were loaded with 4 mL of 7% hypertonic saline instead of albuterol solution and nebulizers were operated for 10 min [33].

As expected, we found significant differences in nebulizer performance when units were evaluated without and with breathing simulation. Solution output, sputtering time, inspiratory filter, expiratory filter, and drug out were lower while nebulizer mass was higher when data obtained without breathing simulation were compared to data obtained with breathing simulation. Our data confirm previous reports that showed the relevance of choosing breathing simulation parameters to evaluate nebulizer performance [38]. Our data highlight the importance of standardization in the evaluation of nebulizer performance [39]. Our data also match previous observations from Ho et al., who reported an increase in drug output associated with the increase in entrained flow (0 to 20 L/min) through a PARI LC Star nebulizer during evaluation with simulated breathing [43]. Leung et al. reported similar findings with entrained flows varying from 0 to 35 L/min [25].

Previous studies evaluating nebulizer performance after repeated sterilization did not use breathing simulation [33,34]. Our data, as shown in Figure 7, agree with O’Callaghan et al., who reported an overestimation of drug output (Sodium cromoglycate) of 24, 26, and 50% in three different nebulizer/compressor systems when using solution output instead of biochemical drug measurement [36]. Tandon et al., similarly to our data, reported an overestimation of drug output when solution output was used as a surrogate (≈81%) [37]. The use of the gravimetric method or standing cloud technique overestimates nebulizer performance. This can be further confounded by not using breathing simulation [44].

One strength of this study is that we completed 180 cycles of sterilization, which is equivalent to daily sterilization over the 6 months of use recommended for the device. One limitation of this study was that the arm that did not use breathing simulation only completed 60 cycles. Another limitation of this study was that we did not measure aerosol characteristics. Future studies should include this evaluation. Another limitation was that nebulizers were only operated during the scheduled evaluation. In addition, we tested a single brand of a breath-enhanced nebulizer and a single brand of a baby bottle sterilizer. However, the choice was made because it is the most used and is also the one that had previously published data to compare. Future studies should include similar testing methodologies extended to other disinfection methods and nebulizer brands and types.

## 5. Conclusions

Thermal disinfection with a baby bottle sterilizer did not alter the performance of a reusable breath-enhanced nebulizer. These results support the continuation of the use of this thermal disinfection method. Our data highlighted the importance of using appropriate methodology to evaluate nebulizer performance. Two important principles were validated. Our results confirmed that nebulizer testing without breathing simulation underestimated its performance. Finally, our data confirmed that calculating drug output based on solution output resulted in its overestimation. 

## Figures and Tables

**Table 1:** Experimental design.

*n*	Cleaning/Disinfecting Cycles	0	5	10	15	20	30	60	90	120	150	180
5	Cleaning-Breathing simulation	X	X	X	X	X	X	X	X	X	X	X
	Disinfecting-Breathing simulation	X	X	X	X	X	X	X	X	X	X	X
5	Cleaning-No breathing simulation	X	X	X	X	X	X	X				
	Disinfecting-No breathing simulation	X	X	X	X	X	X	X				

X = performed.

**Table 2:** Outcome variables obtained with breathing simulation.

Variable	Solution Output (mL)		Nebulizer Mass (%)		Sputtering Time (s)		Inspiratory Filter (%)		Expiratory Filter (%)	
Cycles	CLEAN	STER	CLEAN	STER	CLEAN	STER	CLEAN	STER	CLEAN	STER
0	1.29 ± 0.12	1.22 ± 0.11	65.7 ± 4.9	68.1 ± 5.5	3.6 ± 0.5	3.8 ± 0.8	16.8 ± 2.6	18.0 ± 2.6	5.2 ± 1.1	6.5 ± 1.4
5	1.09 ± 0.17	1.14 ± 0.15	74.5 ± 5.2	70.0 ± 4.7	3.8 ± 1.3	4.6 ± 1.3	11.8 ± 2.4	12.6 ± 2.2	4.5 ± 0.8	5.4 ± 1.4
10	1.43 ± 0.05	1.37 ± 0.05	60.9 ± 0.7	63.4 ± 2.5	6.0 ± 0.7	6.0 ± 0.7	20.1 ± 1.4	17.5 ± 1.1	7.0 ± 1.1	7.0 ± 0.7
15	1.29 ± 0.25	1.21 ± 0.02	65.4 ± 9.4	64.5 ± 2.0	4.4 ± 0.9	5.2 ± 0.4	16.2 ± 4.6	17.2 ± 0.7	6.0 ± 2.1	6.0 ± 0.6
20	1.43 ± 0.08	1.23 ± 0.15	58.9 ± 2.1	67.2 ± 5.7	6.0 ± 1.0	4.4 ± 0.9	21.5 ± 1.7	18.6 ± 3.3	7.8 ± 1.2	7.4 ± 1.2
30	1.54 ± 0.08	1.35 ± 0.09	56.4 ± 2.6	63.0 ± 3.1	5.8 ± 0.4	5.4 ± 0.5	21.2 ± 1.7	17.9 ± 2.2	8.1 ± 1.5	6.8 ± 1.3
60	1.38 ± 0.09	1.33 ± 0.05	58.4 ± 4.2	60.3 ± 2.4	4.6 ± 0.5	5.0 ± 0.0	22.4 ± 1.4	21.2 ± 2.1	7.8 ± 1.6	8.2 ± 0.7
90	1.32 ± 0.05	1.36 ± 0.06	58.2 ± 1.6	58.2 ± 2.8	4.6 ± 0.9	4.4 ± 0.5	20.4 ± 0.9	20.8 ± 1.9	6.8 ± 1.4	6.4 ± 1.3
120	1.27 ± 0.05	1.30 ±.0.05	60.2 ± 2.7	60.7 ± 2.5	4.4 ± 0.5	4.8 ± 0.4	19.1 ± 1.0	19.4 ± 2.2	6.8 ± 1.0	6.1 ± 0.5
150	1.33 ± 0.05	1.32 ± 0.07	58.6 ± 1.7	58.5 ± 2.6	4.2 ± 0.4	4.8 ± 0.4	20.0 ± 1.4	20.9 ± 1.5	7.2 ± 1.1	6.7 ± 0.8
180	1.31 ± 0.11	1.33 ± 0.07	59.7 ± 3.9	59.5 ± 2.5	4.4 ± 0.5	4.8 ± 0.8	19.5 ± 2.0	19.4 ± 1.2	6.3 ± 1.1	6.6 ± 1.0

CLEAN = cleaning and STER = sterilization.

## Data Availability

Data are contained within the article.
